# Jefferson Medical College

**Published:** 1844-01-13

**Authors:** H. T. Child


### JEFFERSON MEDICAL COLLEGE.

### CLINIC OF PROFESSOR MÜTTER.

*December* 6, 1843.

(Reported by H. T. Child.)

#### A CASE OF SEROUS ENCYSTED TUMOUR OF THE SUPERIOR MAXILLARY BONE.

A. B. aged about 13 years, of a fine constitution and good general health, about twelve months since perceived, for the first time, a distinct swelling of the upper maxillary bone, which gave him no pain, however, and hence caused no complaint. Since that period the swelling has continued to increase, until it now equals in size a hen's egg, and occasions the swelling of the face and also projects across the roof of the mouth. It is scarcely at all painful, and he seeks relief in consequence of the deformity, and also for fear of the disease becoming worse. When we press the tumour its walls *yield and crackle* like *parchment*, and as soon as the pressure is *removed return by their own elasticity to their original position.* Externally the surface is smooth, and no adhesions exist between it and the adjacent cheek. Its colour is *mottled*, being red in some spots, and greyish or whitish in others. The alveolar process seems to be sound, and there is *but one carious tooth on the side of the tumour.*

The lymphatic glands in the neighbourhood are healthy, and in short there is no trace of any constitutional disturbance.

*Remarks.*—The case before us, gentlemen, is evidently one of those termed by Hawkins and others, "*serous encysted tumour*" of bone, which Dupuytren called "*fibro-cellular tumour of bone,*" and to which the terms *Spina ventosa*, *Wind-ball*, *Egg-shell tumour*, &c., have been given by the older authorities.

*Causes.*—The causes of such growths are often obscure, but frequently they arise from blows and injuries of various kinds inflicted on the bones, giving rise to inflammation and its products, and when met with in the jaw may usually be traced to some disease of the *teeth or alveolar process.*

That disease of the roots of the teeth characterised by the growth of little cysts attached to the fang at the bottom of the alveolar process, and which are often removed along with the tooth, perfectly sound and entire, is especially the cause in many cases of the formation of a tumour of the kind under consideration. When the cyst is on the *side* of the fang, it often makes its way by progressive absorption through the bone to the gum, and there forms a tumour similar in shape, colour, and consistence, to a common parulis, and ultimately discharges spontaneously by ulceration or remains stationary for a length of time, producing more or less local inconvenience. If opened it often heals without difficulty; but occasionally it remains fistulous, discharging pus of a healthy character, and requiring for its cure the entire destruction of the cyst.

Where the disease commences in the alveolar process itself, the cyst either forms a tumour on the inner or outer surface of the gum, or takes a direction upwards (if in the superior maxillary bone,) until it reaches the antrum, and there either empties itself or gradually enlarging, gives rise to a tumour of large size—the walls of the antrum yielding from pressure until they become almost of the consistence of parchment. It is not improbable, too, that this form of tumour may originate in the establishment of certain entozoa in the cellular tissue of the bone. We must be careful, however, not to confound this tumour with that described by Sir C. Hawkins, as the "hydatid encysted tumour," in which the disease may be traced, in every instance, to the presence of hydatids.

*Bones most liable to be attacked.*—Although any of the bones may be attacked with this disease, some are much more liable than others. The *upper* and *lower jaw bones*, the *extremities* of the *long bones*, the *vertebræ*, and the *bones of the fingers and toes*, are of this class. The predisposition seems to be dependent, for the most part, on their higher degree of organization and the looseness of their tissue; for in no case does the disease *originate* in the *compact* texture of the bone.

*Effects upon the bone.*—The change in the structure of the bones attacked with this form of disease, is very interesting, though we cannot trace precisely the agents operating in the production of this change. It appears, however, that the cellular tissue first, and then the compact immediately around the cyst, are removed in part or entirely, by a process analogous to progressive absorption in other organs; but as the cyst reaches the outer laminæ, nature, in order to protect herself from worse evils, deposits a smaller quantity of earthy matter than usual in the parts pressed upon; and thus the animal portion being in excess, the bone expands and becomes thinner and thinner until it resembles parchment. Were it not for this provision, ostitis, caries, necrosis, or some malignant disease of the bone would result from the continued irritation kept up by the presence of the cyst.

*Size and shape of the tumour.*—These tumours vary much in shape and size: generally they are oval or round, occupy oue *side*, or the *entire circumference* of a long bone, when these bones are attacked and are rigidly *circumscribed;* not spreading off into the surrounding parts, as tumours of a different character are wont to do. In size they vary from that of a garden pea to masses of several feet in diameter.

*Age most liable.*—We have no data upon which to found a statement here: I have seen the disease in children, adults, and old men; but so far as my own observation extends, it is much more frequently met with in young persons.

*Symptoms.*—The phenomena characteristic of serous encysted tumour of bone are modified by the location of the disease, its duration, size, and shape But there are certain general symptoms by which its presence may usually be inferred. Where, for instance, we find a tumour *indolent in its character*, but *slightly if at all painful*, *presenting no evidence of malignant action*, *smooth on the surface*, *elastic even when of small size*, *crackling under the pressure of the finger*, *circumscribed and occupying one side of a bone*, *or involving regularly its entire circumference*, it is highly probable that it belongs to the serous encysted class.

Sometimes the tumour, when small, is firm and hard, like other osseous tumours.

*Diagnosis.*—This form of tumour has been, in consequence of its elasticity, confounded with medullary sarcoma, or fungus hematodes; but the crackling of the walls under pressure, together with the history of the case, and the general condition of the patient, will be sufficient to distinguish one from the other. It has also, in its early stages, been mistaken for exostosis. It has also been mistaken for "osseous aneurism," but the pulsation in the latter disease will be sufficient to indicate its presence. In all doubtful cases puncture or an attempt to puncture with a trochar or grooved needle, will give us its true character, and should be invariably had recourse to.

*Prognosis.*—When the disease is confined to a small bone or a cavity readily reached, as the antrum, where it is of a small size, and the cause producing it of such a nature as to be readily eradicated, the prognosis is favourable; for there is no reason to believe that the constitution is involved, or that there exists any local malignant action. But where the case is of long standing, the tumour deep seated, or very large, and the cause more or less permanent in its nature, the loss of the organ involved, the loss or disorder of an important function, or possibly the loss of life itself, may be the result.

*Dissection.*—When we examine this tumour carefully with the knife we find that it is composed of a thin shell of bony, fibrous, or cartilaginous matter, within which in some cases there exists a number of *spines of bone*, passing in every direction, either attached at both ends to the walls, or by one only, the other projecting into the centre of the cavity and hanging like a stalactite; in other cases there are none of these. These spines of bone divide the cavity into a series of cells, communicating with each other, lined by a delicate membrane, and filled with fluids of different kinds. For example, we may have a thin watery or slightly mucilaginous liquid, of a reddish, opaque, or yellowish hue, or it may be transparent; sometimes it is thicker, like jelly or steatomatous matter; again, it may be almost solid, looking like cheese, but easily separated from the cyst. The soft parts covering the tumour are mostly condensed and thinned, or perhaps partially absorbed or displaced by the pressure, but we find in them no traces of disease.

*Treatment.*—Various methodsi of treatment have been proposed for the relief of ths disease; some of these are harsh and cruel, while others have been feeble and inefficient.

The following are the general means now employed, to the exclusion of all others; but it is obvious that the *size*, *location*, and *nature* of the *contents* of the tumour must determine which of these plans is best adapted to the case.

1st. *Simple puncture of the cyst and the evacuation of its contents.* This operation will often succeed in removing the disease where the *tumour is small*, of *recent origin*, and *filled with thin fluid*, which is readily drawn off. I have in two or three cases, when the upper jaw was the seat of the disease, opened the cyst by the extraction of a tooth, or when it extended into the antrum, by puncturing this cavity with a small trochar. The swelling generally subsides in a few days and the patient recovers without the use of any other remedy.

2d. *Puncture followed by compression.* In small tumours, about the fingers or bones of the forearm, it is stated that the evacuation of the contents of the cyst, and then the compression of a bandage firmly applied, has answered a very good purpose. I have never tried this method myself, but from the fact that the walls of the cyst are flexible, am disposed to recommend its employment.

3d. *Puncture followed by the seton.* In obstinate cases where the fluid is secreted after each puncture, and when the tumour is tolerably large, a very good plan is to open it freely and then introduce a piece of lint which will act as a seton, and cause the secretion to change from a thin serum to healthy pus in a few days. Every day the lint must be removed, and the cyst washed out with some mild injection—warm water, or flaxseed tea will answer very well.

The walls of the tumour gradually contract under the influence of interstitial absorption, while the cavity is filled up, in part, by granulations. This method is exceedingly useful when the upper jaw is the seat of the disease.

4th. *Puncture followed by injection of stimulating fluids, breaking up the spines with a probe, the application of caustic, or the actual cautery.* These plans of treatment are only justifiable in cases of long standing, when all the other means have failed, and the lining membrane is so callous that it is necessary to destroy it entirely before healthy granulations can form. There is always great danger of necrosis of the adjacent bones, and sloughing of the neighbouring soft parts, from these powerful applications. Much benefit, however, occasionally results from the injection of *weak* solutions of sulphate of zinc or copper, or nitrate of silver where the lining membrane is more dense and callous than usual.

5th. *Opening the tumour, removing its semi-solid contents, destroying its surface, and pressure.* This operation is, of course, confined to one variety of the disease, and is always attended with the risk of exciting inflammation and all its consequences. I have known it succeed in one case, but I would much rather remove the whole tumour or amputate the limb, than resort to it again.

6th. *Excision of the tumour, or amputation of the limb on which it has formed.* Where the tumour is large, more or less solid, the adjacent bones diseased, and the patient old or feeble, if any thing is done, the disease should be removed entirely, either by excision of the part attacked, or by amputation.

In the case before us, I shall first puncture the tumour in order to ascertain the character of its contents and then decide as to the subsequent course of treatment. I now introduce the trochar, and you perceive a few drops of gelatinous fluid, so viscid as scarcely to flow, escapes through the canula. From this circumstance and the large size of the tumour, I shall make a free opening into the antrum, so that the fluid may readily escape. I will first extract the diseased tooth, and probably its fang may reach the cavity of the cyst.

It does not, as you see. I will, therefore, take out the next which I find is also diseased, although I did not detect this before. I am firmly resisted here, and it seems that anchylosis has taken place between the tooth and the alveolar process: we will divide the process with the cutting forceps, in order to save the patient pain, and also be certain of opening the antrum. Now it comes away, and the contents of the tumour are discharged. We shall order warm flaxseed washes—keep the patient at home for a few days—restrict him to vegetable diet; and should inflammation supervene, leeches, purges, and the antiphlogistic plan of treatment will be employed. The result of the treatment will be reported to you.

Then were two cases of hernia. One of these occurring in a child about 4 years old, was double inguinal hernia, which distended the scrotum until it reached more than half way down to the knees, causing an immense enlargement of this sac. Professor M. remarked that as the hour had expired, he would not detain us any longer than to reduce the hernia—which was readily done—and apply the double truss. The other case—a brother of the last patient—had single hernia of the same variety, and also had the truss applied.

